# Systematic Insight of Resveratrol Activated SIRT1 Interactome through Proximity Labeling Strategy

**DOI:** 10.3390/antiox11122330

**Published:** 2022-11-25

**Authors:** Tian Su, Zhengyi Zhang, Xiao Han, Fei Yang, Zhen Wang, Ying Cheng, Huadong Liu

**Affiliations:** 1 Center for Mitochondrial Biology and Medicine, The Key Laboratory of Biomedical Information Engineering of Ministry of Education, School of Life Science and Technology, Xi’an Jiaotong University, Xi’an 710049, China; 2 School of Health and Life Sciences, University of Health and Rehabilitation Sciences, Qingdao 266071, China

**Keywords:** SIRT1, resveratrol, proximity labeling, RanGap1, ROS

## Abstract

SIRT1 functions by regulating the modification of proteins or interacting with other proteins to form complexes. It has been widely studied and found to play significant roles in various biological processes and diseases. However, systematic studies on activated-SIRT1 interactions remain limited. Here, we present a comprehensive SIRT1 interactome under resveratrol stimulation through proximity labeling methods. Our results demonstrated that RanGap1 interacted with SIRT1 in HEK 293T cells and MCF-7 cells. SIRT1 regulated the protein level of RanGap1 and had no obvious effect on RanGap1 transcription. Moreover, the overexpression of Rangap1 increased the ROS level in MCF-7 cells, which sensitized cells to resveratrol and reduced the cell viability. These findings provide evidence that RanGap1 interacts with SIRT1 and influences intracellular ROS, critical signals for mitochondrial functions, cell proliferation and transcription. Additionally, we identified that the SIRT1-RanGap1 interaction affects downstream signals induced by ROS. Overall, our study provides an essential resource for future studies on the interactions of resveratrol-activated SIRT1. There are conflicts about the relationship between resveratrol and ROS in previous reports. However, our data identified the impact of the resveratrol-SIRT1-RanGap1 axis on intracellular ROS.

## 1. Introduction

The sirtuin family encodes class III histone deacetylases (HDACs), which are homologous to the *Saccharomyces cerevisiae* transcription regulator Sir2 [1]. Mammalian sirtuins SIRT1-7 are nicotinamide adenine dinucleotide (NAD)^+^-dependent deacetylases [2] that are widely distributed in cells with different subcellular localizations. SIRT1, SIRT6 and SIRT7 are mainly located in the nucleus. SIRT3, SIRT4 and SIRT5 are mitochondrial proteins, and SIRT2 is primarily located in the cytoplasm [2]. Most studies have demonstrated that the members of this family play essential roles in a variety of cellular processes such as modulating lifespan [3], apoptosis [4], mitochondrial biosynthesis [5], lipid metabolism [6], fatty acid oxidation [7], cellular stress responses [8], insulin secretion [9], and aging [10]. Among these seven sirtuins, SIRT1 is the most widely studied.

One of the most well-known functions of SIRT1 is extended lifespan [3]. In earlier studies, calorie restriction improved metabolic health and extended lifespan through the activation of SIRT1 [3]. As a deacetylase, SIRT1 plays crucial roles in many biological processes causing various modifications of histone and nonhistone protein acetylation statuses, such as the forkhead transcription factors (FOXOs) [11], Ku70 [12], the tumor suppressor p53 [13] and peroxisome proliferator-activated receptor γ coactivator-1α (PGC-1α) [14]. Several studies have shown that SIRT1 can regulate apoptosis by deacetylating p53 and inhibiting p53-dependent transcription during cellular stress [15]. By changing the acetylation status of Ku70, SIRT1 regulates DNA repair [12]. SIRT1 is involved in various diseases. Previous studies reported that SIRT1 could improve healthy ageing and protect against metabolic-syndrome-associated cancers [16]. By regulating insulin secretion and protecting pancreatic β-cells, SIRT1 protects cells from oxidative stress and inflammation [17] and has become a therapeutic target in the prevention of type 2 diabetes. The studies also reported that SIRT1 could regulate cancer cell growth and apoptosis [18]. The inhibition of SIRT1 decreases the Matrix metalloproteinase 2 (MMP2) and FOXO3a expression in MCF-7 cells which participate in apoptosis and tumor invasion [18]. Recent research demonstrated that the activation of SIRT1 could regulate PGC-1α signaling to improve Drp1-mediated mitochondrial dysfunction in manganese-induced nerve damage in mice [19]. In addition, resveratrol, a natural SIRT1 activator, also plays important roles as an antioxidant in mitochondrial function and longevity [20]. However, the functions of resveratrol are still controversial. Although resveratrol could enhance TNFα-induced apoptosis, it also inhibits angiotensin-II-induced apoptosis [21]. Additionally, it is reported that resveratrol could not robustly extend the life span in model organisms through activating SIRT1 [21]. While performing complex biological functions, SIRT1 interacts with histone or nonhistone substrates which are important targets for many diseases and participates in intracellular signal transduction and protein modification. Therefore, it is of great significance to study the interactome of SIRT1 to understand its biological processes and develop new strategies for the treatment and prevention of diseases.

Studies based on protein–protein interaction (PPI) networks could be helpful in understanding the substrates and functional regulation of proteins of interest (POIs). In earlier reviews, researchers mapped the PPI network of SIRT1 and predicted its basic molecular mechanism, describing the complex regulation of this protein [22]. Several studies have also reported the interactome of SIRT2, SIRT6 and SIRT7. In 2012, researchers identified proteins and complexes associated with SIRT7 [23]. Some research revealed that their study expanded the knowledge of SIRT2 cytoplasmic functions to define its previously unrecognized involvement in intracellular trafficking pathways [24]. However, the research of the SIRT1 interactome under activating conditions is still lacking. In addition, traditional detection and enrichment methods were used in previous studies, which are unable to capture transient and weak interactions that fully reflect the actual situation in cells. Therefore, proximity labeling was used to better understand dynamic changes in the interactome of activated SIRT1.

Proximity labeling based on an engineered peroxidase 2 (APEX2) was developed in 2015, which is an upgrade of APEX technology and has higher activity than the previous one [25]. Compared with other proximity labeling methods, the entire process of APEX2 labeling requires only 1 min to label, which could better capture dynamic change in the proteome. In the presence of hydrogen peroxide (H_2_O_2_), APEX2 catalyzes biotin-tyramide or biotin-phenol (BP) to generate short-lived free radicals, such as phenolic arylazide derivatives [26] or tyramine derivatives [27]. These radicals can be covalently attached to electron-rich amino acid side chains and react covalently with specific amino acids, including tyrosine (Tyr), tryptophan (Trp), cysteine (Cys), and histidine (His) [28,29,30]. Therefore, this method could capture transient and weak interactions in living cells.

Here, we establish, to our knowledge, the first proteomics-based dynamic network of protein interaction changes following SIRT1 activation. We used APEX2-based proximity labeling to systematically reveal the interactome changes and validated the prominent interactions. Our findings identified and predicted new targets in the regulation of SIRT1 and provided a unique perspective on the likely means of regulating SIRT1 functions in health and disease processes.

## 2. Materials and Methods

### 2.1. Materials

H-DMEM (12800-058) was purchased from Gibco (Grand Island, NE, USA). EX-527 (49843-98-3-5 mg) was purchased from MCE (Princeton, NJ, USA). Resveratrol (R107315-25 g), dithiothreitol (DTT, D104860-25 g) and iodoacetamide (I105563-25 g) were purchased from Aladdin (https://www.aladdin-e.com/, accessed on 10 November 2017). Bicinchoninic acid (BCA) kit (#23225), Pierce^TM^ Streptavidin Magnetic Beads (88816) and V5-Tag antibody (R960-25) were purchased from Thermo (Waltham, MA, USA). BP (41994-02-9) and horse radish peroxidase (HRP)-streptavidin (#RABHRP3) were purchased from Sigma (St. Louis, MO, USA). IgG (A7028) was purchase from Beyotime (Shanghai, China). Protein A/G PLUS-Agarose (sc-2003), G3BP1 (sc-365338), GAPDH (sc-47724), Erk (sc-514302), c-Fos (sc-166940) and PGC1-α (sc-517380) were purchased from Santa Cruz (Dallas, TX, USA). SIRT1 (IF3) mouse mAb (#8469S), RanGap1 (#36067S), β-actin (#3700S), Src (#2108S), Phospho-p44/42 MAPK (Erk1/2) (#4370S), Drp1 (#14647S), Mfn1 (#14739S), Parkin (#32833S), PINK1 (#14647S), p53 (#6946S) and TFAM (#8076S) were purchase from Cell Signaling Technology (Boston, MA, USA). TRIzol (9109) and Prime Script RT Master Mix (Perfect Real Time) (RR036A) was purchased from Takara (Dalian, China).

### 2.2. Cell Culture

Human embryonic kidney (HEK) 293T cells and MCF-7 cells, acquired from ATCC (Manassas, VA, USA), were cultured in H-DMEM supplemented with 10% fetal bovine serum (FBS), 100 U/mL penicillin, and 100 μg/mL streptomycin. Cells were treated with 20, 30, 40 or 50 μM resveratrol or 1 μM EX-527 for 24 or 48 h. Then, proteins were extracted for Western blotting. All cells were maintained at 37 °C in a 5% CO_2_ humidified environment.

### 2.3. Plasmid Constructions and Generation of shRNA Expression Cells

To generate a recombinant SIRT1-APEX2 plasmid for APEX2 labeling and RanGap1-HA plasmid for RanGap1 overexpression, the primers were used as below:

5′-ATTTGCGGCCGCATGGCGGACGAGGCGGCCCT (Forward of SIRT1-APEX2)

5′-CGCGGATCCGCTGATTTGTTTGATGGATAGTTCATG (Reverse of SIRT1-APEX2)

5′-CTAGCTAGCATGGCCTCGGAAGACATTGCCAAGCTGGCAGAGACACTTGCCAA (Forward of RanGap1-HA)

5′-GGAATTCCTAAGCGTAGTCTGGGACGTCGTATGGGTAGACCTTGTACAGCGTCT (Reverse of RanGap1-HA)

HEK 293T cells and MCF-7 cells were transfected with SIRT1-APEX2 plasmid or RanGap1-HA plasmid using Lipofectamine reagent for 48 h. The cells transfected with APEX2 plasmid and pcDNA3.1 plasmid were negative control.

To generate SIRT1-knockdown and RanGap1-knockdown cell lines, HEK 239FT cells were transfected with pCMV-dR8.2 and pCMV-VSV-G, and indicated expression plasmids (SIRT1 knockdown plasmids or RanGap1 knockdown plasmids) using Lipofectamine reagent for 48 h (pLkO.1 plasmid as a negative control). Lentivirus-containing medium was collected and used for infection of HEK 239FT cells and MCF-7 cells in the presence of 6 μg/mL polybrene and then selected in the medium containing 2 μg/mL puromycin until cells could stably survive. The primers were used as below:

5′-CCGGGCGGGAATCCAAAGGATAATTCTCGAGAATTATCCTTTGGATTCCCGCTTTTTG (Forward of SIRT1-shRNA)

5′-AATTCAAAAAGCGGGAATCCAAAGGATAATTCTCGAGAATTATCCTTTGGATTCCCGC (Reverse of SIRT1-shRNA)

5′-CCGGGAAGATGCTAAAGATGTGATTCTCGAGAATCACATCTTTAGCATCTTCTTTTTG (Forward of RanGap1-shRNA)

5′-AATTCAAAAAGAAGATGCTAAAGATGTGATTCTCGAGAATCACATCTTTAGCATCTTC (Reverse of RanGap1-shRNA)

### 2.4. APEX2 Labeling and Preparation of Cell Lysates

The method used to obtain proteins tagged by biotin-phenoxyl radicals is described as follows [31]. HEK 239T cells were cultured in 100 mm plates. When grown to 30% confluency, cells were transfected with SIRT1-APEX2 recombinant plasmid using Lipofectamine reagent. The medium was replaced by H-DMEM after 24 h. After 48 h, cells were incubated with 500 μM BP and 20 μM resveratrol in H-DMEM for 30 min at 37 °C. Then, 1 mM H_2_O_2_ in PBS was directly added to the BP solution. Cells were incubated at room temperature (RT) for 1 min and washed three times with quencher solution (10 mM sodium ascorbate, 5 mM Trolox, and 10 mM sodium azide solution in PBS). Cells for negative control that omit BP and H_2_O_2_ to assess the background signal contributed by nonspecific sticking and for positive control that omit resveratrol.

Cells were lysed in RIPA lysis buffer (50 mM Tris-HCl, pH 7.5, 150 mM NaCl, 0.1% (wt/vol) SDS, 1% (vol/vol) Triton X-100) on ice for 2 min supplemented with 1 mM PMSF. Then, cell lysates were centrifuged for 10 min at 15,000× *g* at 4 °C. The supernatant was collected in a 1.5 mL tube, and the bicinchoninic acid (BCA) kit was used to measure protein concentration. Some samples were taken, and loading buffer was added and then boiled at 95 °C for 10 min for Western blotting and Coomassie blue staining. Other samples were prepared to enrich with Pierce ^TM^ Streptavidin Magnetic Beads.

### 2.5. Samples Alkylation, Protein Enrichment, and on Beads Digestion

In total, 5 μL 200 mM DTT was added to a 100 μL sample and vortexed. Samples were placed at RT for 1 h. 100 μL sample was alkylated by adding 4 μL 1 M IAA and vortexed and placed at RT for 1 h. Then, the remaining IAA was neutralized by adding 20 μL 200 mM DTT to a 100 μL sample. 

Streptavidin beads were washed with 1 mL RIPA lysis buffer twice. Then, samples were incubated with 30 μL streptavidin beads for 1 h at RT on a rotator. Beads were pelleted by a magnetic rack, and supernatants were collected. Then, beads were washed with a series of buffers (1 mL for each wash) to remove nonspecific binders: twice with RIPA lysis buffer, once with 1 M KCl, once with 0.1 M Na_2_CO_3_, once with 2 M urea in 10 mM Tris-HCl, pH 8.0, and twice with PBS. The wash buffers were kept on ice throughout the procedure. 

Trypsin was added to the 8 M Urea and 50 mM NH_4_HCO_3_ buffer. Then, streptavidin beads were incubated with this solution at 37 °C and gently vortexed and spined for 18 h. Digested peptides were desalted as described by this protocol [32]. Two plugs of Empore C18 material were packed into StageTip. StageTips were placed in 1.5 mL tubes using StageTip adapters and washed with 100 μL acetonitrile, followed by centrifuging at 3000× *g* for 3 min at RT. Then, StageTips were equilibrated with 100 μL ddH_2_O supplemented with 0.1% (vol/vol) formic acid and centrifuged. Samples were loaded onto the StageTips and subsequently washed with 100 μL ddH_2_O supplemented with 0.1% (vol/vol) TFA twice. Peptides were eluted into clean 1.5 mL tubes with 100 μL of 50% (vol/vol) acetonitrile supplemented with 0.1% TFA (vol/vol). Solutions were dried by vacuum centrifugation at RT and then dissolved in 30 μL ddH_2_O supplemented with 0.1% (vol/vol) TFA.

### 2.6. LC-MS/MS Analysis

The QE-plus MS was used to analyze samples with a data-dependent acquisition mode. The parameters were set as follows: the capillary temperature was 320 °C, the electrospray voltage was 2.1 kV, the maximum filling time was 120 ms, the automatic gain control target was 1 × 10^6^ ions, the orbital resolving power was 35,000 at 200 *m*/*z* and the quadrupole isolation window was 2 *m*/*z* units [33]. The parallel reaction monitoring (PRM) method was performed to detect precursors [34].

The human database downloaded from UniProt (https://www.uniprot.org/, accessed on 29 March 2019) was used for database searching and identifying by the MaxQuant software. The parameters were set as follows: trypsin digestion, the mass deviation of the maximum fragment ion was 0.5 Da, the mass deviation of the maximum parent ion was 20 ppm, the mass of the maximum peptide segment was 4600 Da, and the shortest peptide segment was 7. Then, the label free quantitation method was used for quantification. The PRM data were filtered using Skyline software and exported in Excel.

### 2.7. Cell Viability Assay

MCF-7 cells were seeded in 96-well plates at a density of 2 × 10^4^ per well for 24 h. Cells were treated with resveratrol at concentrations of 0, 20, 30, 40, and 50 μM for 4 or 24 h. Cell viability was then determined using a 3-(4,5-dimethyl-2-thiazolyl)-2,5-diphenyl-2-H-tetrazolium bromide (MTT) assay [35]. 

### 2.8. Co-Immunoprecipitation (Co-IP)

HEK 293T cells and MCF-7 cells were cultured in 100 mm plates and transfected with SIRT1-overexpression plasmids. After 48 h, cells were lysed by IP lysis buffer with 100 mM PMSF and centrifuged for 10 min at 12,000 rpm at 4 °C. Then, the lysate was incubated with 20 μL Protein A/G PLUS-Agarose and 1 μg IgG at 4 °C for 30 min on a rotator. After that, the lysate was centrifuged for 5 min at 3000 rpm at 4 °C, and the supernatant was transferred into a clean 1.5 mL tube on ice. The supernatant was incubated with 1 μg SIRT1 (IF3) mouse mAb at 4 °C on a rotator. After 1 h, this solution was incubated with 20 μL Protein A/G PLUS-Agarose at 4 °C on a rotator overnight. The solution was centrifuged for 5 min at 3000 rpm at 4 °C, and the supernatant was collected. The pellet was washed with 1 mL PBS 4 times. The 60 μL of 1× loading buffer was added, and samples were boiled at 100 °C for 2–3 min and analyzed by SDS polyacrylamide gel electrophoresis (SDS-PAGE).

### 2.9. RNA Extraction and Real-Time (RT) PCR

TRIzol was used to extract the total RNA of HEK 293T cells and MCF-7 cells, which were treated with resveratrol, overexpressed SIRT1 or knocked down SIRT1. The PrimeScript RT Master Mix was used to reverse transcribe RNA (1 μg) into cDNA. The RT–PCR was performed with Bio-Rad system SYBR Green protocol. The results were calculated using the 2^–ΔΔCt^ method with GAPDH as an internal control. The primers were listed in Table 1. 

### 2.10. Intracellular Reactive Oxygen Species (ROS) Determination

A fluorescent probe dihydroethidium (DHE) was used to detect intracellular ROS. After being treated with 20 μM resveratrol, cells were washed with PBS and incubated with 10 μM DHE for 30 min at 37 °C in the dark. Then, cells were washed with PBS and lysed with lysis buffer (10 mM Tris, 0.1 mM EDTA-2Na, 0.5% TritonX-100 and 150 mM NaCl in ddH_2_O, pH 7.5). Cell lysates were centrifuged for 10 min at 13,000× *g* at 4 °C. The supernatant was collected in a 1.5 mL tube, and the BCA kit was used to measure protein concentration. The fluorescence intensity was measured with a fluorescence spectrometer at 535 nm excitation and 610 nm emission. The ROS level was expressed as the relative fluorescence per microgram of protein (BCA method).

### 2.11. Western Blot Assay

Proteins were separated by 10% or 12% SDS–PAGE at 90 V for 120 min. Then, proteins were transferred to nitrocellulose filter membranes at 300 mA for 100 min. After being blocked with 5% skimmed milk in Tris-buffered saline with Tween (TBST) buffer for 1 h at RT, membranes were washed with TBST 3 times. Then, membranes were incubated with the primary antibodies diluted in TBST buffer containing 1% BSA at 4 °C overnight. The primary antibodies as follows: V5-Tag (1:1000), SIRT1 (1:1000), RanGap1 (1:1000), β-actin (1:2500), horse radish peroxidase (HRP)-streptavidin (1:1000), G3BP1 (1:500), GAPDH (1:2500), Src (1:1000), Erk (1:1000), Phospho-p44/42 MAPK (Erk1/2) (1:1000), c-Fos (1:1000), PGC1-α (1:1000), Drp1 (1:1000), Mfn1 (1:1000), Parkin (1:1000), PINK1 (1:1000), p53 (1:1000) and TFAM (1:1000). After that, the membranes were washed three times with TBST. Then, the membranes were incubated with the secondary antibodies (anti-rabbit or anti-mouse IgG at 1:3000 in TBST containing 1% BSA) for 2 h at RT and washed three times with TBST. The membranes were visualized using an ECL Western blot detection kit and the intensity of the bands was quantified using ImageJ software. 

### 2.12. Statistical Analysis

The Microsoft Office Excel, Skyline (Ver. 21.1), GraphPad Prism 5 statistical software packages and Image J software were used for data quantification and analyses. T-test or one-way ANOVA test were used to determine significance, including * *p* < 0.05, ** *p* < 0.01 and *** *p* < 0.001. The STRING database (https://www.string-db.org/, accessed on 18 August 2019), R software and Cytoscape software were used to plot the heatmap and network. The website KOBAS (http://kobas.cbi.pku.edu.cn/, accessed on 5 September 2021) [36] was used to perform GO analysis. According to the expression level of the differentially expressed genes, a log-based on 2 was used to calculate the Euclidean distance. Then, the hierarchical cluster method was used to obtain the overall clustering of the samples. All experiments were repeated at least three times.

## 3. Results

### 3.1. Construction of SIRT1-APEX2 Plasmid and Identification of Proximity Labeling

**Figure 1:**
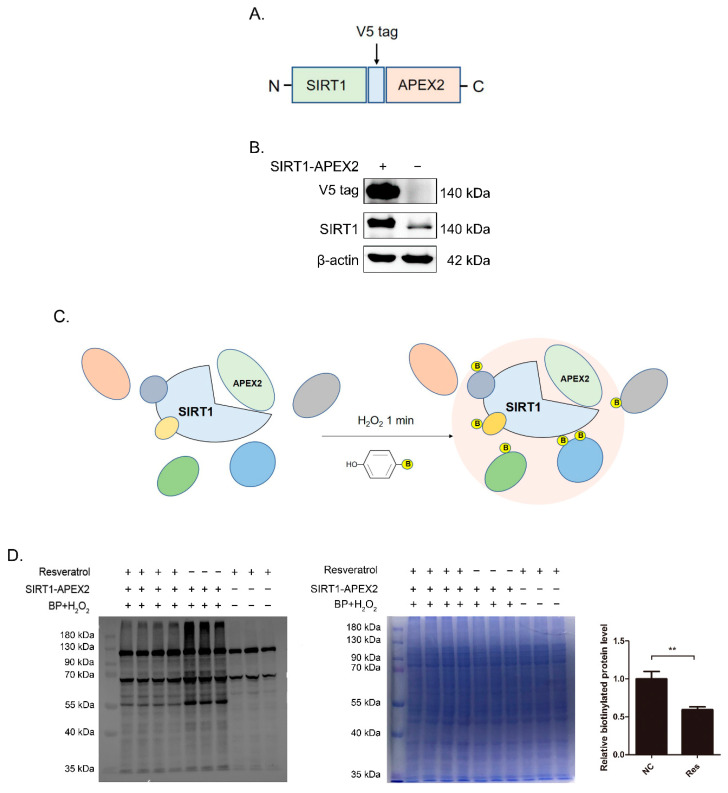
Expression and activity characterization of SIRT1-APEX2 recombinant protein. HEK 293T cells were transfected with SIRT1-APEX2 (>50% transfection efficiency) and labeled. Cells were then lysed, separated by SDS-PAGE and analyzed by blotting with streptavidin-horseradish peroxidase. (**A**) A schematic drawing of the SIRT1-APEX2 constructs. SIRT1 gene (light green) was ligated to 5′ end of APEX2 (pink) with a V5 tag (blue). (**B**) Analysis of recombinant protein expression by SIRT1-APEX2 in HEK 293T cells. (**C**) Scheme showing APEX2-catalyzed biotinylation. Live cells are incubated with biotin-phenol (BP) probe (yellow B = biotin) for 30 min and then treated for 1 min with 1 mM H_2_O_2_ to initiate biotinylation. APEX2 catalyzes the one-electron oxidation of biotin-phenol into a biotin-phenoxyl radical, which covalently tags proximal endogenous proteins. (**D**) Characterization of APEX2 mediated biotinylation of endogenous proteins by streptavidin blotting. Negative controls in which resveratrol, BP and H_2_O_2_ or SIRT1-APEX2 were omitted. The β-actin and Coomassie brilliant blue staining were used as loading controls. The pooled data were shown here as mean ± SEM, and the significant differences from control cells were shown in *p* < 0.01 (**). The independent experiments were repeated at least three times.

To systematically identify SIRT1-associated proteins, we generated HEK 293T cell lines stably expressing SIRT1-APEX2 fusion protein (Figure 1A) that reported the changes in the protein environment triggered by SIRT1 activation. HEK 293T cells were selected because these cells are easily transfected and have been commonly used in proximity labeling studies [29,31]. 

As shown in Figure 1B, Western blotting demonstrated that SIRT1-APEX2 fusion proteins were successfully expressed, and were overexpressed compared with endogenous SIRT1. Figure 1C reveals the principles of APEX2 labeling. Under the catalysis of H_2_O_2_ for 1 min, APEX2 could catalyze BP to generate phenolic arylazide derivatives and covalently bind to specific amino acids such as Tyr, Trp, Cys, and His. Then, we performed streptavidin-HRP blot analysis of the whole cell lysates to validate whether the endogenous proteins were biotinylated by APEX2 (Figure 1D). The groups omitting the SIRT1-APEX2 plasmid, BP and H_2_O_2_ or resveratrol were negative controls. Coomassie brilliant blue staining was used as a loading control. Resveratrol is a natural SIRT1 activator which activates SIRT1 through allosteric regulation [37]. As Figure 1D shows, we observed bands that only appeared in the presence of APEX2 and H_2_O_2_, which spanned a large molecular weight range. Only three bands at 72, 75, and 130 kDa appeared in the negative controls, indicating that they were nonspecific bands. Altogether, these results demonstrated that SIRT1-APEX2 fusion protein has been successfully expressed and induced biotinylation of proteins in the <20 nm range. 

### 3.2. Dynamic Changes in SIRT1 Interactions in Response to Resveratrol

**Figure 2:**
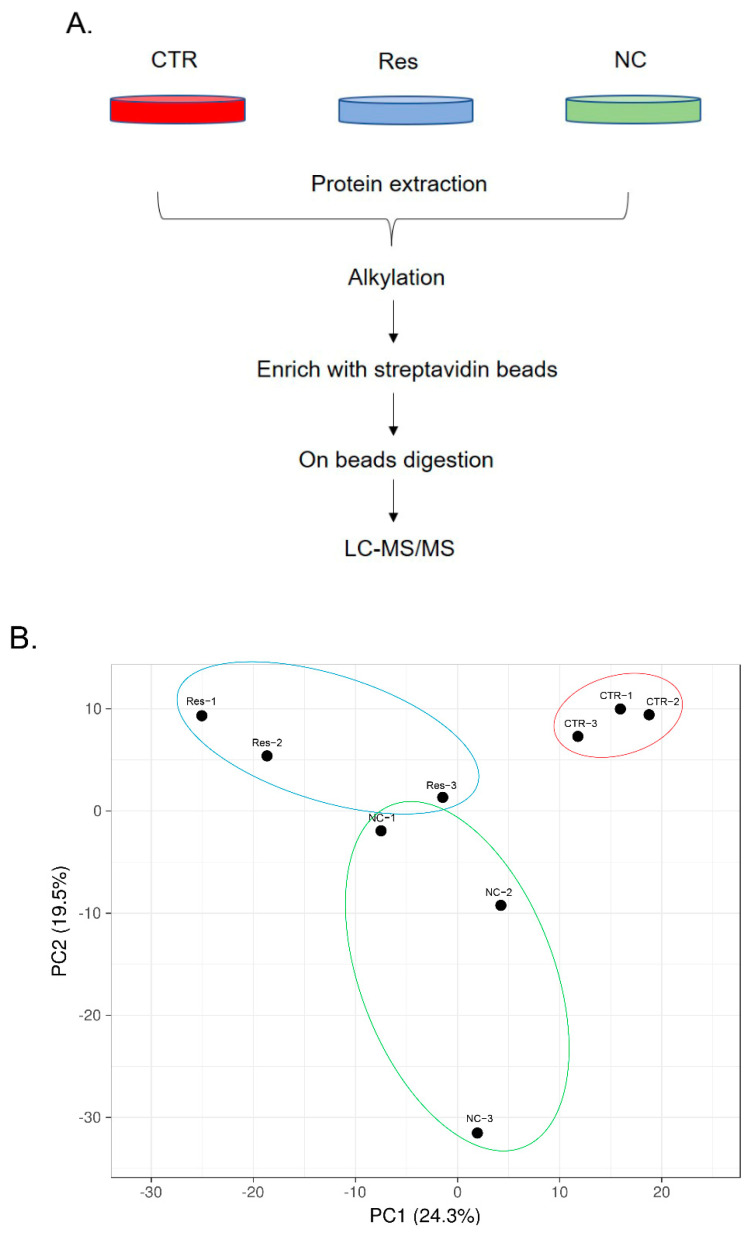

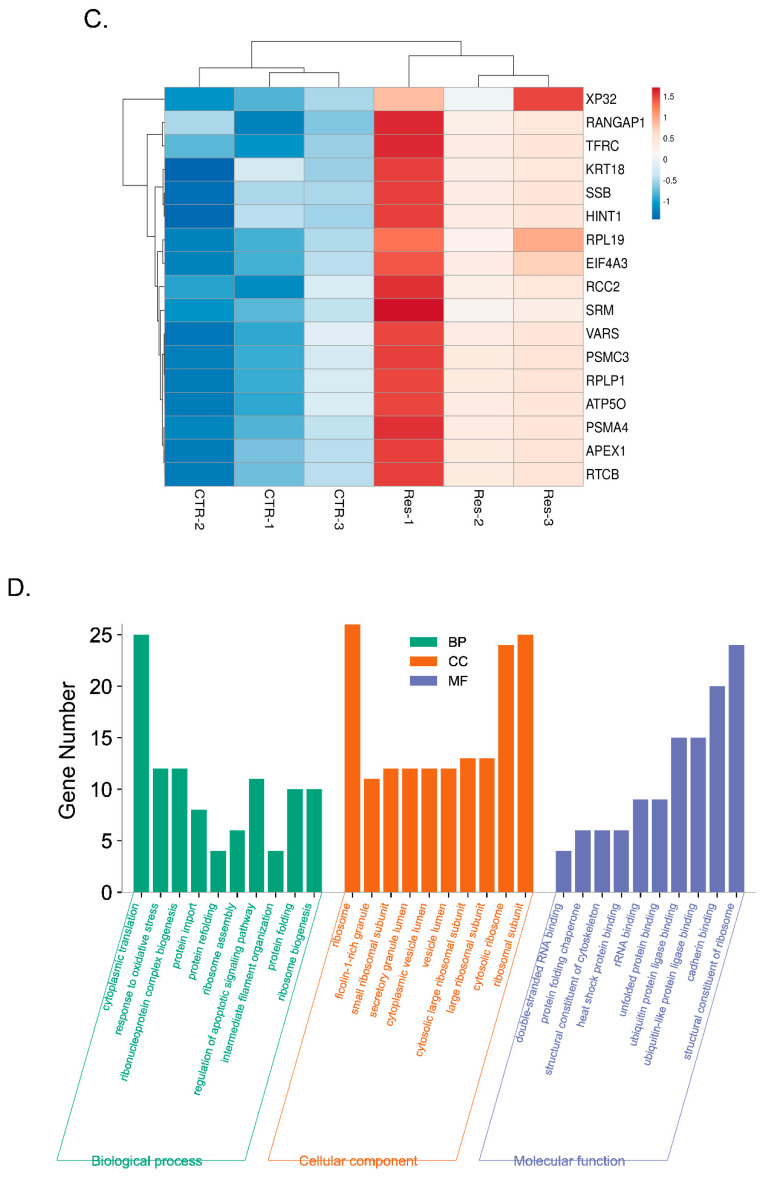

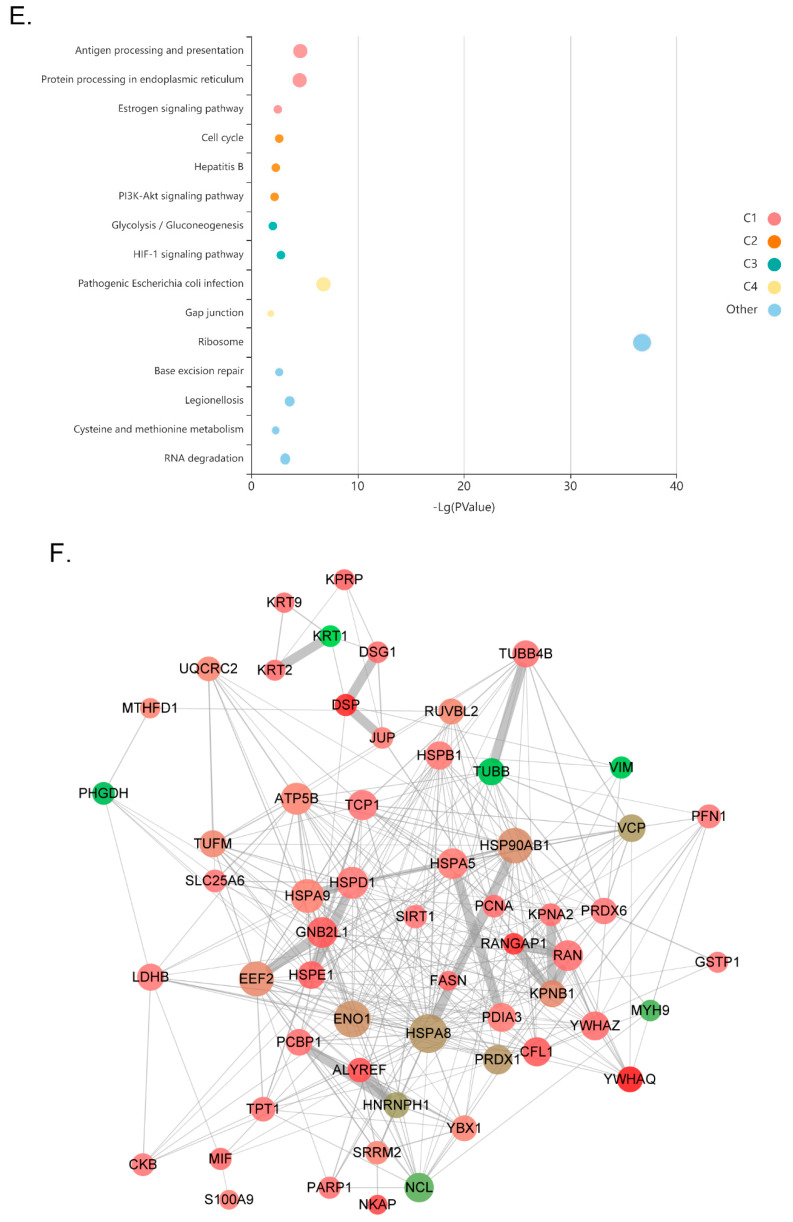
Proteomics analysis of activated SIRT1 interactome. (**A**) Workflow of SIRT1 interactions involving samples preparation and mass spectrometry analysis. (**B**) PCA analysis. SVD with imputation was used to calculate principal components. X and Y axis showed principal component 1 and principal component 2 that explain 24.3% and 19.5% of the total variance, respectively. N = 9 data points. NC: negative control. CTR: control. Res: resveratrol. (**C**) Heatmap. (**D**) Enrichment of the expressed genes at the secondary GO Term in a significantly up-regulated or significantly down-regulated expression. The abscissa was the number of genes. The ordinate was the GO Term. (**E**) Enriched terms visualized in bubble plot. Each bubble represents an enriched function, and the size of the bubble from small to large: [0.05, 1], [0.01, 0.05), [0.001, 0.01), [0.0001, 0.001), [1 × 10^−10^, 0.0001), [0, 1 × 10^−10^). The color of the bar is the same as the color in the circular network, which represents different clusters. For each cluster, if there are more than 5 terms, the top 5 with the highest enrich ratio were displayed. (**F**) Dramatic changes of biotinylated proteins were visualized by string database. The biotinylated protein level reduced by resveratrol was filled in green; the increased level was filled in red. The size of the circle and depth of color indicate the fold changes, the larger circles and deeper colors mean a higher fold change.

We prepared negative control group (NC group: −BP, −H_2_O_2_), control group (CTR group: +BP, +H_2_O_2_, −resveratrol) and resveratrol group (Res group: +BP, +H_2_O_2_, +resveratrol), as shown in Figure 2A. Each group had six biological replicates. Taking advantage of the APEX2 labeling [31], the biotinylated proteins were enriched with streptavidin beads and were digested on the beads with trypsin. Then, LC–MS/MS was performed. 

To validate the quality of the MS detection, PCA and correlation coefficient analysis were performed. As shown in Figure 2B, PCA revealed that the Res groups were clustered together in comparison with the controls, which implies a high reproducibility among these samples. From Figure S1A, we also observed that among ourdata, the highest correlation coefficient of the same biological replicate group could reach 0.99, such as CTR-1 and CTR-2. The correlation coefficient between the treatment group and the control group could be as high as 0.98, such as Res-2 and CTR-3. Furthermore, we found that the Res-1 group had lower values than the other groups; thus, we treated these data with more caution in the subsequent data processing and analysis. Then, we selected proteins that co-occurred in all samples to plot the heatmap (Supplementary Table S1). Figure 2C demonstrates that these proteins were upregulated after SIRT1 activation, suggesting that SIRT1 might function by recruiting proteins to interact or modify them. To present the change trend of the proteome, a volcano plot was generated. By using the cut-off at fold-change > 1.5 and *p* value < 0.05, 9 proteins were upregulated (in red), and 337 proteins were downregulated (in green). The majority did not show dramatic changes (in grey) (Figure S1B).

GO analysis was performed to understand the functions of the biotinylated proteins. Our data clearly showed that approximately 25 genes participated in cytoplasmic translation, which was the most significant biological process (Figure 2D). This phenomenon might be related to SIRT1’s transcriptional regulation function, since SIRT1 deacetylates transcription factors to regulate downstream proteins [38]. Another notable biological process is the response to oxidative stress, including more than 10 genes. Previous studies reported that SIRT1 was a critical regulator in response to oxidative stress [39,40]. In addition, we observed that some genes were involved in ribonucleoprotein complex biogenesis, and they might be structural constituents of ribosomes. As shown in Figure 2D, approximately 15 genes play roles in ubiquitin protein ligase binding and ubiquitin-like protein ligase binding. This result suggested that the activation of SIRT1 might participate in protein degradation. From Figure 2E, we found that approximately 16% of genes were enriched in ribosomes, which is consistent with Figure 2D. Approximately 3% of the genes participated in protein processing in the endoplasmic reticulum, with *p* values ranging from 1 × 10^−10^ to 0.0001. Additionally, we also observed that approximately 1% of genes were involved in the PI3K-Akt signaling pathway, and 2% of genes were related to the cell cycle, with *p* values ranging from 0.0001 to 0.001.

To identify the functional relationship between SIRT1 and the biotinylated proteins, high-confidence interactions were extracted from the STRING database. The results were visualized by Cytoscape as shown in Figure 2F. Upregulated proteins are represented in red, and downregulated proteins are represented in green under resveratrol conditions. From this network, we observed that most proteins were increased, and some proteins have previously been reported in SIRT1 interaction maps [22], such as Y box binding protein 1 (YBX1) and heat shock protein 90 kDa alpha (cytosolic) class B member 1 (HSP90AB1). This result indicated that our data overlap with other data, supporting the reliability of our data. In addition to these proteins that have previously been reported to interact with SIRT1, we also found some proteins related to acetylation, such as lactate dehydrogenase B (LDHB) and Ran. Previous studies reported that SIRT5 interacted with and mediated the deacetylation of LDHB at K329 [41] and SIRT7 deacetylated Ran at K37 to regulate the nuclear export of p65 [42]. We speculated that SIRT1 might affect the acetylation of these proteins. Altogether, we established the first network of SIRT1-activatedinteractions. 

### 3.3. SIRT1 Interacts with RanGap1 and G3BP1

Among the SIRT1 interacting proteins, we focused on Ran GTPase-activating protein 1 (RanGap1) because it was more significant than the other proteins in three independent LC–MS/MS experiments, which had *p* value < 0.05 and fold change > 1.5 (Supplementary Table S2). RanGap1 is a GTPase activator of Ran that plays a role in Ran-mediated nuclear import and export. RanGap1 is located in the cytoplasm, and the SUMO1-RanGAP1 is located in the nuclear pore which can bind to the nucleoporin RanBP2/Nup358 to regulate the cell cycle. Additionally, we validated Ras-GTPase-activating protein SH3 domain-binding protein 1 (G3BP1), which had a *p* value < 0.05 and fold change > 1.5 in an independent LC–MS/MS experiment (Supplementary Table S3 and Figure S1C). G3BP1, which can inhibit tumor cell apoptosis, is usually overexpressed in tumors and cancers, such as MCF-7 cells. 

**Figure 3:**
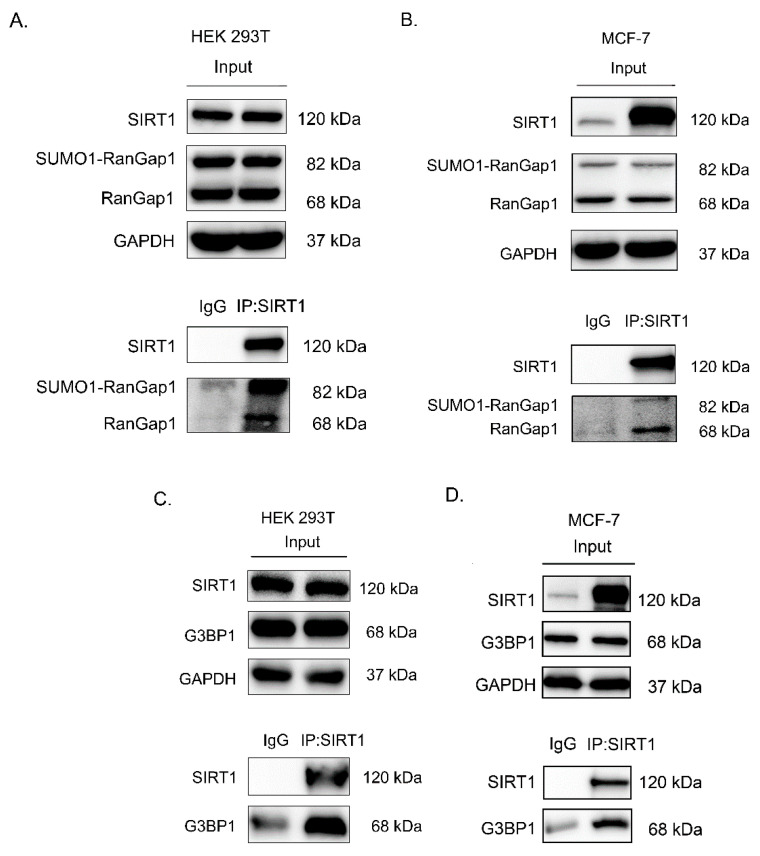
Interaction of SIRT1–RanGap1 and SIRT1–G3BP1 occurs in 2 epithelial cell lines. (**A**,**C**) SIRT1 interacts with RanGap1 and G3BP1 in HEK 293T cells. (**B**,**D**) SIRT1 interacts with RanGap1 and G3BP1 in MCF-7 cells. The independent experiments were repeated at least three times. IgG was used as a negative control.

To validate the interaction between SIRT1 and RanGap1, endogenous RanGap1 was immunoprecipitated with SIRT1 from HEK 293T cells. As shown in Figure 3A, the Western blot analysis showed clear bands in the anti-SIRT1 antibody group and no clear bands in the IgG group. This result suggests that endogenous SIRT1 could interact with RanGap1. To verify whether this interaction also existed in other cell lines which were regulated by SIRT1 activation in our previous report [43], endogenous RanGap1 was immunoprecipitated with SIRT1 from MCF-7 cells. Figure 3B demonstrates the same result as Figure 3A, which implies that SIRT1 interacts with RanGap1 in both HEK 293T cells and MCF-7 cells. As shown in Figure 3C,D, G3BP1 also interacted with SIRT1 in HEK 293T cells and MCF-7 cells. In addition, as shown in Figure S1C, our data displayed a reduction in G3BP1 after activation of SIRT1, which means that resveratrol decreases G3BP1 [44], possibly through SIRT1 regulation. Therefore, these results demonstrated that SIRT1 could interact with RanGap1 and G3BP1, suggesting that SIRT1 might regulate these two proteins.

### 3.4. SIRT1 Regulates RanGap1 Expression

**Figure 4:**
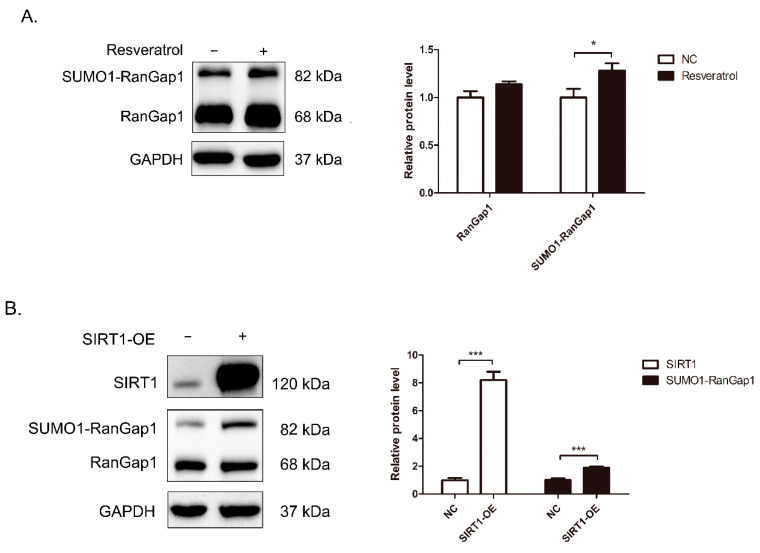

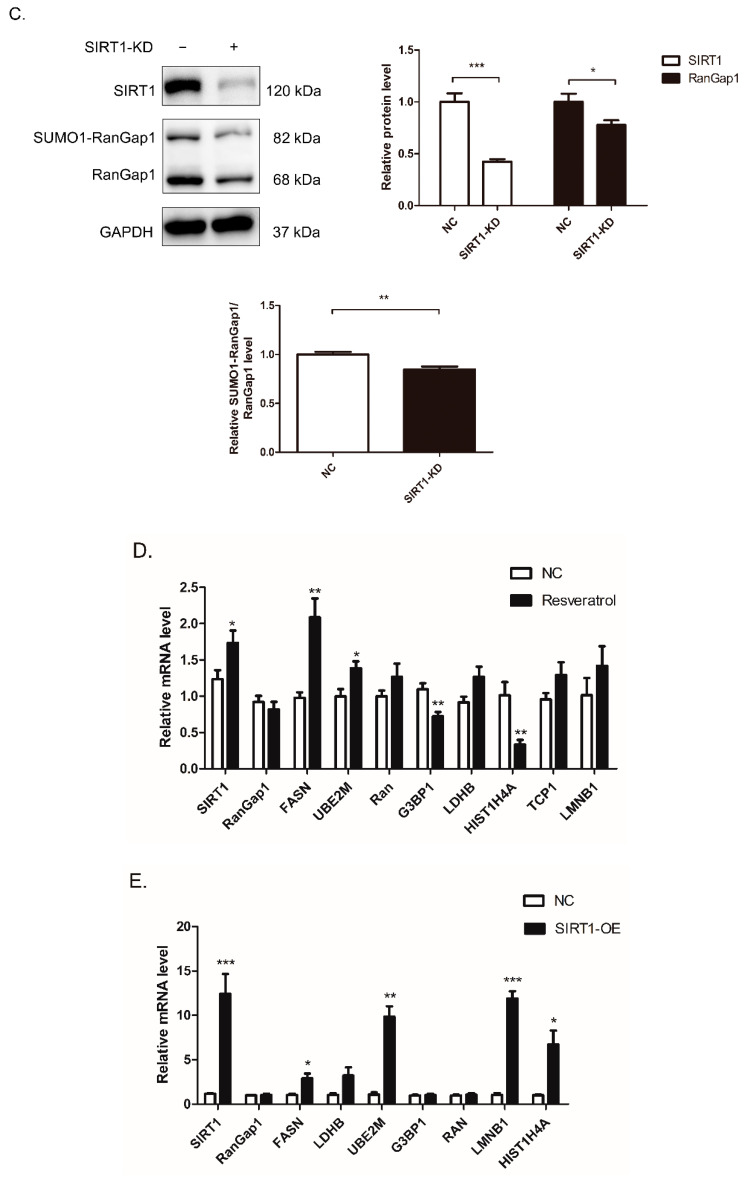

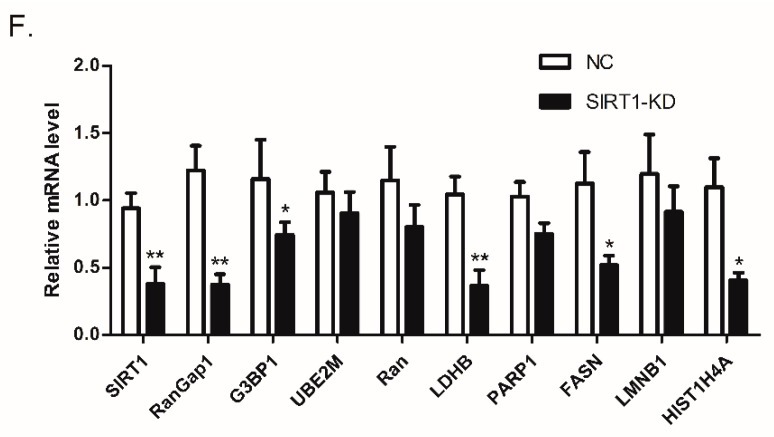
SIRT1 regulates RanGap1 expression. After treating with 20 μM resveratrol for 24 h, or having SIRT1 over-expressed or knocked down in HEK 293T cells, the relative mRNA levels were determined by RT-PCR and the protein levels were detected by Western blot. (**A**) SUMO1-RanGap1 was increased after activation of SIRT1. (**B**) SUMO1-RanGap1 was increased in SIRT1-OE cells. (**C**) RanGap1 was decreased in SIRT1-KD cells. (**D**–**F**) The mRNA levels of SIRT1, RanGap1, G3BP1, FASN, UBE2M, Ran, LDHB, TCP1, LMNB1, HIST1H4A and PARP1 from cells as above. The GAPDH was used as a loading control. The pooled data were shown here as the mean ± SEM, and the significant differences from control cells were shown in *p* < 0.05 (*), *p* < 0.01 (**), and *p* < 0.001 (***). The independent experiments were repeated at least three times.

To verify whether the expression and activity of SIRT1 influence RanGap1, Western blots were performed for protein analysis. As shown in Figure 4A,B, SUMO1-RanGap1 was increased after SIRT1 activation or SIRT1 overexpression in HEK 293T cells. Figure 4C shows reductions in RanGap1 and SUMO1-RanGap1 in SIRT1-KD cells. These results suggested that both the expression and activity of SIRT1 affected RanGap1. 

Then, to confirm whether these changes were caused at the transcriptional level, we extracted RNA from HEK 293T cells that were activated by resveratrol or had SIRT1 overexpressed or knocked down. As shown in Figure 4D–F, the mRNA level of RanGap1 did not appear to be significantly changed in SIRT1-activated and SIRT1-overexpressing (SIRT1-OE) cells. These data provided evidence that SIRT1 activation might play a role in SIRT1-RanGap1 interaction and affects their stability in a mRNA independent manner. However, the mRNA level of RanGap1 was decreased in SIRT1-knockdown (SIRT1-KD) cells, indicating the critical and complicate function of SIRT1. In addition, significant genes were also selected for RT–PCR analysis, as shown in Supplementary Table S3. Fatty acid synthetase (FASN) and UBE2M transcripts were increased in SIRT1-activated and SIRT1-OE cells, and FASN was significantly decreased in SIRT1-KD cells, suggesting that FASN might be regulated by SIRT1 through SIRT1/AMPK signaling [45]. The mRNA level of G3BP1 was reduced in SIRT1-activated cells, which is consistent with a previous report [44,46], and it was decreased in SIRT1-KD cells. Although the mRNA level of LDHB was intensively changed in SIRT1-OE and SIRT1-KD cells, it did not demonstrate an obvious change after the activation of SIRT1, suggesting that LDHB may not be regulated by SIRT1 activity. HIST1H4A was obviously decreased in SIRT1-activated and SIRT1-KD cells and increased in SIRT1-OE cells. Previous studies reported that SIRT1 could affect transcription by regulating acetylation of HIST1H4A [47]. Therefore, activated SIRT1 regulates many genes’ transcription, but not RanGap1. 

### 3.5. RanGap1 Affects Antioxidant Capacity and Mitochondrial Functions

We observed that some proteins were involved in oxidative stress in the GO analysis (Figure 2D). Our data (Figure 4D) also demonstrated that the activation of SIRT1 could increase the expression of SUMO1-RanGap1. It has been reported that SIRT1 affects reactive oxygen species (ROS) and mitochondrial function [48], which regulates cancer cell growth and apoptosis, such as MCF-7 cells. The activator resveratrol has been reported to increase intercellular ROS, thus showing high cytotoxicity and mediating apoptosis of MCF-7 cells [49]. However, resveratrol also reduced ROS and effectively inhibited the apoptosis of cardiomyocytes and mitochondrial oxidative damage in myocardial ischemia and reperfusion injury models [50], showing a protective effect on cardiomyocytes. Therefore, we wondered what the impact of RanGap1 on ROS is. 

**Figure 5:**
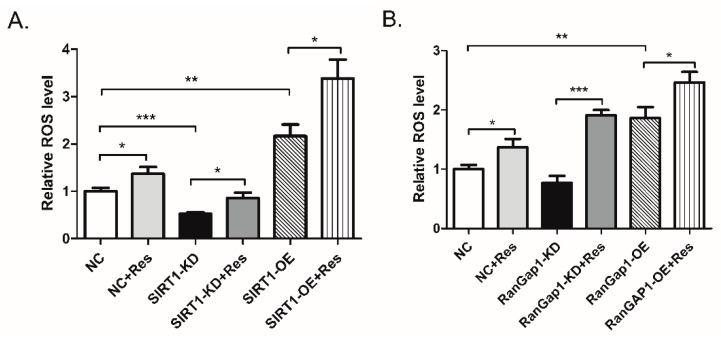

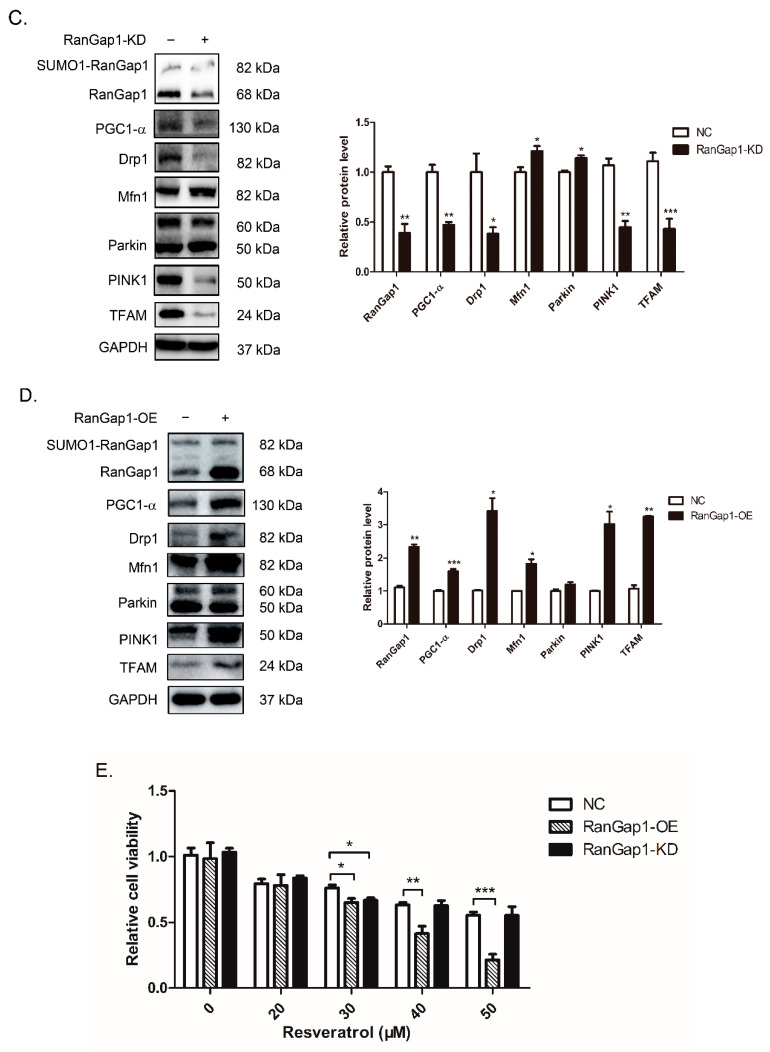
RanGap1 affects antioxidant capacity and mitochondrial functions. (**A**) The ROS level was decreased in SIRT1-KD cells and increased in SIRT1-OE cells. (**B**) The ROS level was increased in RanGap1-OE cells and did not have obvious changes in RanGap1-KD cells. (**C**) PGC1-α, Drp1, PINK1 and TFAM were decreased, and Mfn1 and Parkin were increased in RanGap1-KD cells. (**D**) PGC1-α, Drp1, Mfn1, PINK1 and TFAM were increased in RanGap1-OE cells. (**E**) The cell viability was significantly decreased in RanGap1-OE cells under treating with 30 to 50 μM resveratrol compared to control cells. DMSO was used as negative control. The GAPDH was used as a loading control. The pooled data were shown here as the mean ± SEM, and the significant differences from control cells were shown in *p* < 0.05 (*), *p* < 0.01 (**), and *p* < 0.001 (***). The independent experiments were repeated at least three times.

We detected intracellular ROS levels by using DHE, a ROS fluorescent probe, to confirm whether RanGap1 is related to ROS. Figure 5A clearly shows that the ROS level was decreased in SIRT1-KD cells and increased in SIRT1-OE cells, suggesting the SIRT1 expression could affect the ROS level. After treatment with resveratrol, the ROS level was increased in both cell lines, indicating that resveratrol may cause the accumulation of ROS in cancer cells. As shown in Figure 5B, the ROS level was significantly increased in RanGap1-OE cells, although no apparent change in RanGap1-KD cells was observed. This result suggests that the RanGap1 overexpression could cause oxidative damage to MCF-7 cells by the accumulation of ROS. After resveratrol stimulation, we observed that the ROS level was increased in RanGap1-KD and RanGap1-OE cells. Altogether, these results indicated that RanGap1 expression might affect intracellular ROS. 

Previous studies have reported that SIRT1 can regulate mitochondrial functions by affecting ROS [48,51], which are primarily produced in mitochondria and can cause oxidative damage [5]. In our data, we found that the expression of SIRT1 and RanGap1 affected ROS (Figure 5A,B). Therefore, we detected some indicator proteins in mitochondria and wondered whether the expression of RanGap1 would affect mitochondrial functions such as mitochondrial biogenesis and oxidative damage. As shown in Figure 5C,D, PGC-1α and transcription factor A of mitochondria (TFAM), which are vital regulating factors for mitochondrial biogenesis, were obviously decreased in RanGap1-KD cells and increased in RanGap1-OE cells. Drp1 and Mfn1 are mitochondrial dynamin-related proteins that are involved in mitochondrial fission and fusion. As shown in Figure 5C,D, we found that Drp1 was significantly decreased in RanGap1-KD cells and increased in RanGap1-OE cells. In contrast, Mfn1 was increased in both RanGap1-KD and RanGap1-OE cells, but it was more obvious in RanGap1-OE cells. These results suggested that RanGap1 might participate in mitochondrial fission and fusion. Loss of function of PTEN-induced putative kinase 1 (PINK1) failed to recruit Parkin to mitochondria, which was reported as a cause of mitochondrial autophagy dysfunction and parkinsonism in humans [52]. Figure 5D showed that PINK1 was increased in RanGap1-OE cells, suggesting that the overexpression of RanGap1 could cause mitochondrial dysfunction and induce mitochondrial autophagy. However, Parkin did not show any obvious change in Figure 5C,D, indicating that RanGap1 might mediate mitochondrial autophagy in a Parkin-independent way. Altogether, these data indicated that RanGap1 might play a vital role in mitochondrial biogenesis, fission and fusion and autophagy.

The oxidative stress and mitochondrial dysfunction could affect cell viability. To verify whether RanGap1 could cause damage to cells through affecting mitochondrial functions, we detected the viability of RanGap1-KD and RanGap1-OE cells under different concentrations of resveratrol, which could reduce the cell viability and promote apoptosis in MCF-7 cells. As shown in Figure 5E, the MTT assay indicated that resveratrol decreased cell viability with a dose-dependent manner in RanGap1-KD and RanGap1-OE cells and caused dramatic damage at a relatively high concentration (50 μM). As shown in Figure 5E, we observed that RanGap1-KD cells did not have any apparent changes compared with control cells during resveratrol treatment. However, resveratrol caused more dramatic damage to RanGap1-OE cells than the other cells. Under 30 μM resveratrol stimulation, the viability of RanGap1-OE cells was significantly decreased compared with that of control cells, and the viability of RanGap1-KD cells also showed a reduction. Thereafter, the viability was decreased rapidly with a dose-dependent manner in RanGap1-OE cells. However, the viability of RanGap1-KD cells did not show a significant change compared with control cells at higher concentrations of resveratrol. These results suggested that RanGap1-OE cells could be more sensitive to resveratrol, which might cause damage to MCF-7 cells.

### 3.6. SIRT1 and RanGap1 Participate in the Src/Erk/c-Fos Pathway

**Figure 6:**
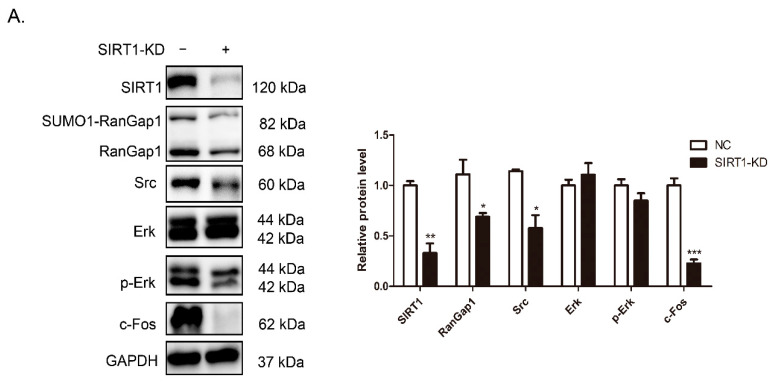

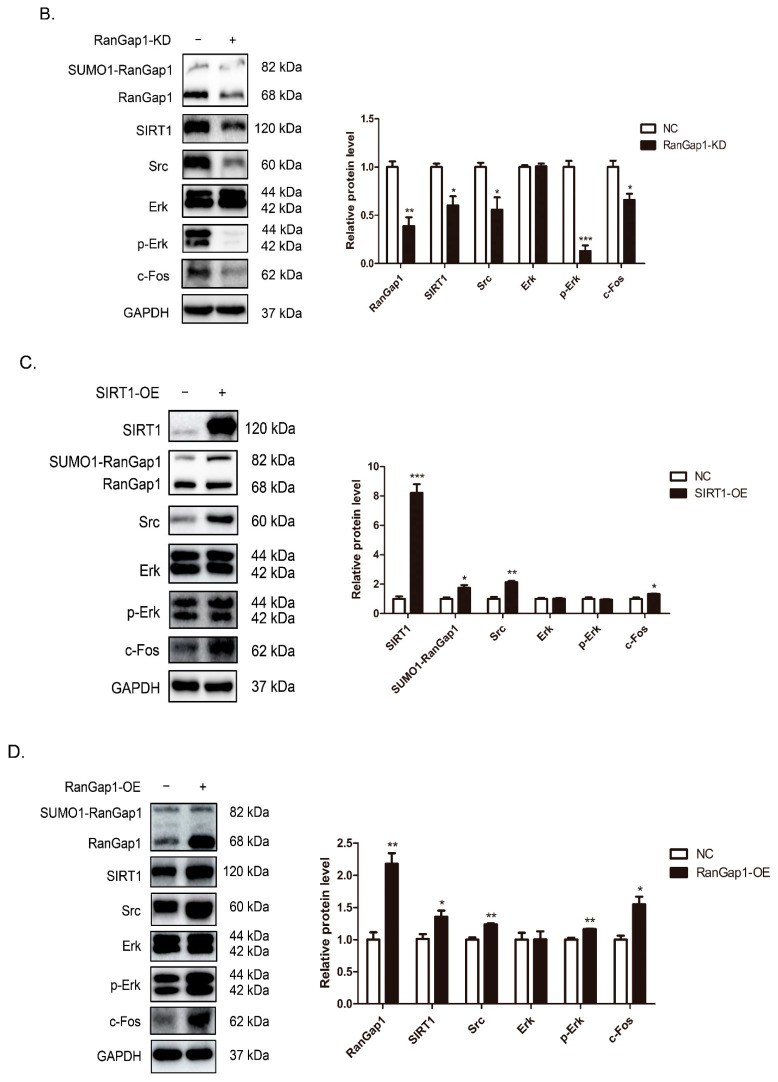
SIRT1 and RanGap1 have effects on Src/Erk/c-Fos pathway. Overexpression and knockdown of SIRT1 and RanGap1 were established in MCF-7 cells, and the protein levels were detected by Western blot. (**A**) RanGap1, Src and c-Fos were decreased in SIRT1-KD cells. (**B**) SIRT1, Src, p-Erk and c-Fos were decreased in RanGap1-KD cells. (**C**) SUMO1-RanGap1, Src and c-Fos were increased in SIRT1-OE cells. (**D**) SIRT1, Src, p-Erk and c-Fos were increased in RanGap1-OE cells. The GAPDH was used as a loading control. The pooled data were shown here as the mean ± SEM, and the significant differences from control cells were shown in *p* < 0.05 (*), *p* < 0.01 (**), and *p* < 0.001 (***). The independent experiments were repeated at least three times.

Previous studies reported that Src plays an important role in oxidative stress-induced phosphorylation [53]. As shown in Figure 5A,B, we observed that SIRT1 and RanGap1 could affect ROS. Therefore, we wondered whether SIRT1 and RanGap1 play roles in some functions by regulating Src. We established SIRT1 and RanGap1 overexpression and knockdown MCF-7 cell lines and performed Western blotting for protein analysis. As shown in Figure 6A, we observed that RanGap1 was decreased in response to a reduction in SIRT1, indicating that SIRT1 could affect the expression of RanGap1 in MCF-7 cells. In addition, we found that Src, p-Erk and c-Fos were obviously decreased in SIRT1-KD cells (Figure 6A), suggesting that SIRT1 could affect transcription by regulating Src expression. Consistently, Figure 6B also demonstrates the reduction of Src, p-Erk and c-Fos in response to RanGap1 knockdown. Since c-Fos is a transcription factor downstream of the Erk pathway, RanGap1 might have additional transcriptional regulatory functions. 

We checked these proteins in SIRT1-OE and RanGap1-OE cells. Src and c-Fos were significantly increased after overexpression of SIRT1 or RanGap1 (Figure 6C,D). Although p-Erk was increased in RanGap1-OE cells, it did not show a significant change in SIRT1-OE cells. All of these data further support the hypothesis that SIRT1 and RanGap1 could affect c-Fos expression through the Src/Erk/c-Fos pathway.

## 4. Discussion

Mammalian SIRT1 has been confirmed to take part in a series of critical cellular processes and pathways in recent studies and it may function as a deacetylase and regulator by modifying or interacting with substrates. Among its various functions, studying the interactome of SIRT1 and revealing its detailed mechanisms would be helpful for understanding its important functions in diseases and cancers. Resveratrol is one of the most widely studied natural activators of SIRT1, and it acts as a mitochondrial nutrient, affecting mitochondrial function. Previous studies have shown various findings about the interactome or acetylome of SIRT1 [22,54], providing us with evidence that may help in understanding SIRT1’s function. Nevertheless, knowledge of the activated SIRT1 interactions is lacking. Therefore, it is crucial to identify the interacting proteins of activated SIRT1 to more fully understand its function in the cellular process. In this study, we presented a proteomics-based analysis of activated SIRT1 protein interactions, established a network of activated SIRT1 binding partners, and revealed the vital role of RanGap1 in regulating transcription and mitochondrial function. 

We performed the first comprehensive proteomic analysis of activated SIRT1 interactions by proximity labeling. Previous studies of SIRT1 interaction often identify single target protein and lack systematic research. A few systematic studies of SIRT1 only analyze data from databases that others have already reported, which could not find new interactions. Or the traditional methods such as IP–MS was used to study the interaction, which might fail to identify the weak and transient interactions. Resveratrol activates SIRT1 through allosteric regulation which is a transient process [37]. Traditional methods might be unable to capture this dynamic change and reflect the actual cell situation. Therefore, we provide a systematic study of the activated SIRT1 interaction by proximity labeling, discover and identify novel proteins that interact with SIRT1. Compared with some traditional labeling methods, proximity labeling could capture the weak and transient interactions that fully reflect the actual situation in cells. APEX2-based labeling is one of the proximity labeling methods and the whole labeling process requires 1 min, which is the fastest labeling among all proximity labeling methods. Compared with the first-generation APEX, the APEX2 higher expressed in cells and the sensitivity and catalytic activity are greatly improved. Therefore, our research is more likely to find these proteins that have weak and transient interactions, which might become novel target of SIRT1 and help in understanding SIRT1 regulation and functions in tumors.

Some proteins that we found were interacting with SIRT1 have been reported in previous studies, such as YBX1 and HSP90AB1 [22]. In that previous study, they obtained experimental data reported in protein databases to obtain SIRT1 first-order interaction maps and an average path length [22], which denotes the average number of steps along the shortest paths for all possible pairs of network nodes [22]. Among the proteins in this network, YBX1 showed the shortest length of 1.64, which means it had the closest distance to SIRT1 and a strong interaction with SIRT1. Similarly, HSP90AB1 and HSPA5, which appeared in our MS data, also demonstrated an average path length of 1.66. We could relate parts of the data in this network to our MS data, including parts of the top 30 best-connected nodes such as YBX1, HSP90AB1, HSPA5, RPS3, RUVBL2, RPL23, and SLC25A6, which appeared in the network. In addition, proteins involved in SIRT1 deacetylation, such as the NFAP gene, were also identified. It has been reported that SIRT1 can deacetylate NF-kappa-B-activating protein (NF-κB) and suppress stimuli-induced NF-κB activation [55]. This phenomenon means that some identified proteins might be directly or indirectly involved in the acetylation regulation of SIRT1. Previous studies have reported that SIRT1 can regulate transcription by deacetylating histones. Therefore, we think that these proteins might participate in the transcription. GO analysis also seems to provide some evidence that a lot of genes in the activated-SIRT1 interaction network were related to ribosomes, which are associated with protein translation. We speculated that the activation of SIRT1 regulates transcription and cause changes on protein translation.

RanGap1 was one of the most significant proteins in our MS data. Early studies identified RanGap1 as a GTPase activator of Ran and it converted cytoplasmic GTP-bound Ran to GDP-bound Ran [56], which is essential for nucleocytoplasmic transport [57] and mitosis [58]. In nucleocytoplasmic transport, substrate forms a complex with receptor to pass through nuclear pore to nucleus. Receptors are returned to cytoplasm by binding with Ran-GTP. RanGap1 hydrolyzed Ran-GTP to Ran-GDP releases receptors. RanGap1 is modified by SUMO1, and it forms the SUMO1-RanGap1 complex located at the nuclear pore. Additional reports linked RanGap1 to the cell cycle [58], transcription [59] and apoptosis [60]. Our observation that SIRT1 interacts with RanGap1 provides further evidence in support of SIRT1 having a role in regulating RanGap1. RanGap1 might be a substrate of SIRT1 or associated with SIRT1 activation and verified this by regulating the expression or activation of SIRT1. By comparing the top 12 SIRT1 deacetylation sequences with RanGap1 sequences, we found a similarity at K524 and speculated that SIRT1 might regulate acetylation of RanGap1 at this site. Previous studies also reported that K524 of RanGap1 could be acetylated [61].

One of the most widely studied roles of RanGap1 is nucleocytoplasmic transport, and SUMO1-RanGap1 can form a complex with RanBP1 to play roles in nuclear import and export. In our research, we found that RanGap1 affected ROS and mitochondrial function in MCF-7 cells, revealing novel functions of RanGap1. Intracellular ROS measurements demonstrated that the ROS level was influenced by RanGap1 expression, which gives us the confidence to explore whether RanGap1 affects mitochondrial function since mitochondria are the major source of endogenous ROS. The detection of mitochondrial indicator proteins demonstrated that RanGap1 affects mitochondrial function. PGC-1α and TFAM are indicator proteins of mitochondrial biogenesis [62]. It was previously reported that the overexpression of PGC-1α induced the expression of mitochondrial genes and increased the number of mitochondria [62]. In our study, we found that RanGap1 could induce the expression of PGC-1α and TFAM, which might cause changes in the number of mitochondria and lead to mitochondrial dysfunction. Intracellular ROS accumulation and mitochondrial dysfunction both cause damage to cells. The effects of RanGap1 on ROS and mitochondrial function imply that RanGap1 might become a novel target in the preventive and treatment on tumors and diseases, especially in breast cancer and mitochondrial diseases. We also found RanGap1 affected the transcription through Src/p-Erk/c-Fos pathway. By knocking down and overexpressing RanGap1 and SIRT1, we showed that RanGap1 and SIRT1 modulate the expression of Src, p-Erk, and c-Fos. Combined with our previous evidence that SIRT1 could regulate the RanGap1 expression, we speculated that SIRT1 might affect the Src/Erk/c-Fos pathway by regulating RanGap1. Another possibility is that SIRT1 interacts with RanGap1 and formats a complex to regulate the Src pathway. Of course, we could not completely exclude the possibility that in this experimental system, Src/Erk/c-Fos pathway was regulated through another pathway. However, we proved that RanGap1 affects the expression of Src, Erk, and c-Fos.

Our data provide evidence of resveratrol-SIRT1-RanGap1 axis in MCF-7 cells, but the exact same result in cells might not be expected in animal studies or clinical trials. Many controversial studies have been reported. Although resveratrol could improve mitochondrial function and plays a role in diseases such as type 2 diabetes and metabolic syndrome in cell lines and animal experiments, previous studies also reported that resveratrol could not robustly extend life span in model organisms through activating SIRT1 [21]. Clinical data suggested that resveratrol benefits type 2 diabetes, metabolic syndrome and nonalcoholic fatty liver diseases, such as improved insulin resistance, body weight and inflammation markers [63]. It was also reported that the magnitude of the effect for resveratrol was trivial in clinical treatment [63]. Due to the sample volume, there is not enough evidence to support a clear answer of the resveratrol roles in the clinical treatment of these diseases. Additionally, the effects of resveratrol are cell type dependent. Consistent with the previous report [49], we identified that resveratrol increases ROS and shows high cytotoxicity of MCF-7 breast cancer cell. However, it has also been reported that resveratrol reduces ROS and protects cardiomyocytes from mitochondrial oxidative damage and apoptosis [50]. Furthermore, the working concentrations of resveratrol varied in many reports. Some reports suggest that resveratrol activates SIRT1 at 11 μM and has no obvious effect at 100–200 μM in cells [64]. However, some experiments showed that resveratrol fails to activate SIRT1 and even reverses the effect at higher concentrations (>50 μM) in U2OS cells [64]. Even higher doses were used in animal models than in cells. It is reported that mice treated with resveratrol were given 100 mg kg^−1^ in vehicle (1% CMC) by oral gavage as well as daily until the experimental endpoint (day 4) [65]. In summary, the resveratrol-SIRT1 activation might play different roles depending on doses, cell types and experimental models. To fully understand the mechanism, activated SIRT1 interactome might need to be verified in specific system.

## 5. Conclusions

In conclusion, our study establishes the proteomics-based dynamic network of protein interaction changes following SIRT1 activation and expands the current knowledge of SIRT1 cellular functions. Specifically, we identified RanGap1 and G3BP1 as proteins that interact with SIRT1 and verified that SIRT1 regulates RanGap1 expression. In addition, this study expands the known cellular functions of RanGap1, which was reported to participate in nucleocytoplasmic transport in previous research. Our results demonstrate that RanGap1 affects antioxidant capacity and the response to resveratrol, and might affect mitochondrial function, including mitochondrial biogenesis, fission and fusion, and autophagy in MCF-7 cells. Breast cancer is often referred to as the “pink killer”, and its incidence ranks first among female malignant tumors. Our findings suggest a possibility that RanGap1 might become the target in prevention and treatment on tumors. Furthermore, we speculated that RanGap1 might be involved in transcription, regulated by SIRT1 through the Src/Erk/c-Fos pathway. Altogether, our findings provide important knowledge about the interactions of activated SIRT1 and suggest that RanGap1 affects transcription and mitochondrial function in MCF-7 cells, meaning that RanGap1 might become a novel target in the prevention and treatment of tumors and diseases.

## Figures and Tables

**Table 1:** Primers used for RT–PCR.

Accession Numbers	Genes	Forward	Reverse
NM_001142498.2	SIRT1	TAGCCTTGTCAGATAAGGAAGGA	ACAGCTTCACAGTCAACTTTGT
NM_002883.4	RanGap1	CACGCCCTCACGGAAGATTC	CTGGGCTATCAGCACGGAG
NM_001256799.3	GAPDH	ACAACTTTGGTATCGTGGAAGG	GCCATCACGCCACAGTTTC
NM_198395.2	G3BP1	CGGGCGGGAATTTGTGAGA	TCTGTCCGTAGACTGCATCTG
NM_003969.4	UBE2M	GGAAGCCAGTCCTTACGATAAAC	CGTTCTGCTCAAACAGCCG
NM_002300.8	LDHB	CCTCAGATCGTCAAGTACAGTCC	ATCACGCGGTGTTTGGGTAAT
NM_006325.5	RAN	TCTGGCTTGCTAGGAAGCTCA	GCTGGGTCCATGACAACTTCT
NM_030752.3	TCP1	CCAGCCACGCTATCCAGTC	TCAAGGCAAGCAATTTTTGCAT
NM_004104.5	FASN	AAGGACCTGTCTAGGTTTGATGC	TGGCTTCATAGGTGACTTCCA
NM_005573.4	LMNB1	GAAAAAGACAACTCTCGTCGCA	GTAAGCACTGATTCCATGTCCA
NM_001618.4	PARP1	TCTGAGCTTCGGTGGGATGA	TTGGCATACTCTGCTGCAAAG
NM_003540.4	HIST1H4F	AACGCATTTCGGGCCTCATT	GCGCGTAGACAACATCCATTG

## Data Availability

The data used in the present study are available on request from the corresponding author.

## Supplementary Materials

The following supporting information can be downloaded at: https://www.mdpi.com/article/10.3390/antiox11122330/s1, Figure S1: MS data analysis; Table S1: The Z-score of proteins that co-occurred in the 6 groups; Table S2: The Z-score of proteins in the 1–3 groups; Table S3: The intensity of proteins in 4–6 groups that *p* < 0.05 and fold change > 2.
