# Supplementary material for: Systematic Insight of Resveratrol Activated SIRT1 Interactome through Proximity Labeling Strategy
Source: Antioxidants (Basel). 2022 Nov 25;11(12):2330. doi: 10.3390/antiox11122330 (PMC9774693; doi:10.3390/antiox11122330)
Supplement: Supplementary file 1 [file antioxidants-11-02330-s001.zip › Fig. S1.pptx]

## Slide 1
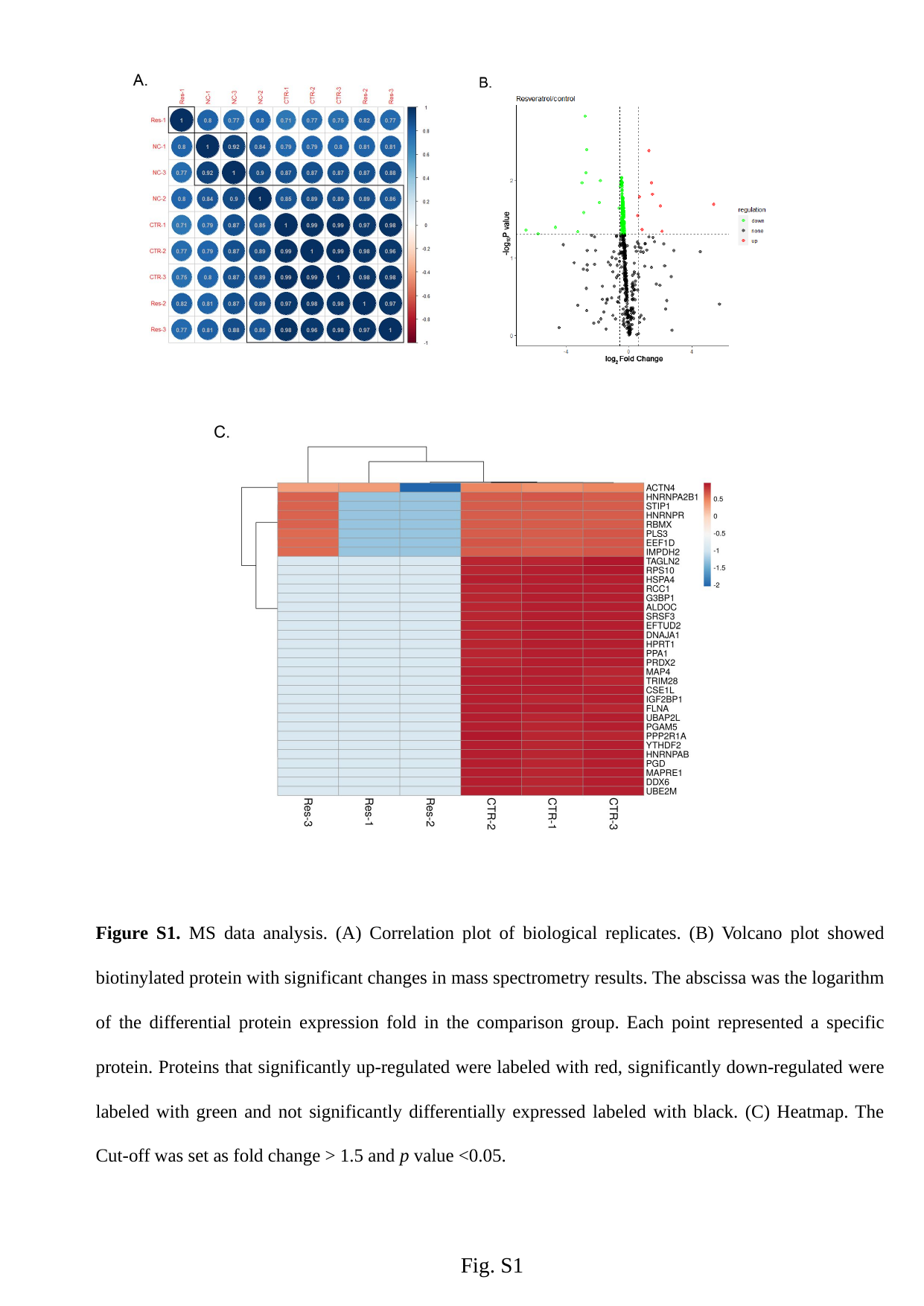

Figure S1. MS data analysis. (A) Correlation plot of biological replicates. (B) Volcano plot showed biotinylated protein with significant changes in mass spectrometry results. The abscissa was the logarithm of the differential protein expression fold in the comparison group. Each point represented a specific protein. Proteins that significantly up-regulated were labeled with red, significantly down-regulated were labeled with green and not significantly differentially expressed labeled with black. (C) Heatmap. The Cut-off was set as fold change > 1.5 and p value <0.05.
Fig. S1
